# Dose–Response Association of Metformin with Parkinson’s Disease Odds in Type 2 Diabetes Mellitus

**DOI:** 10.3390/pharmaceutics14050946

**Published:** 2022-04-27

**Authors:** Kuang-Hua Huang, Ya-Lan Chang, Shuo-Yan Gau, Tung-Han Tsai, Chien-Ying Lee

**Affiliations:** 1 Department of Health Services Administration, China Medical University, Taichung 40402, Taiwan; khhuang@mail.cmu.edu.tw (K.-H.H.); dondon0525@gmail.com (T.-H.T.); 2 Department of Pharmacology, Chung Shan Medical University, No. 110, Sec. 1, Jianguo N. Rd., Taichung 40201, Taiwan; cshd141@csh.org.tw; 3 Department of Pharmacy, Chung Shan Medical University Hospital, Taichung 40201, Taiwan; 4 School of Medicine, Chung Shan Medical University, Taichung 40201, Taiwan; sixsamurai.shien15@gmail.com

**Keywords:** Parkinson’s disease, metformin, diabetes mellitus, cumulative defined daily dose

## Abstract

Background. Studies have demonstrated that patients with diabetes mellitus who receive metformin have a lower risk of developing Parkinson’s disease (PD). However, studies have also suggested that metformin may increase the risk of PD. In this study, we investigated whether metformin use was associated with the risk of PD in type 2 diabetes mellitus (T2DM). Methods. In this population-based cross-sectional study, patients with T2DM diagnosed between 2001 and 2018 were enrolled. We categorized these patients as metformin users or nonusers. Participants below 50 years old were excluded. Two models were employed to evaluate the associations of metformin exposure and use intensity with PD after 3 and 5 years of follow-up. Results. Patients with T2DM who received <300 cumulative defined daily doses (cDDD) of metformin and those with metformin use intensity of <10 DDD/month had respective odds ratios (ORs) for PD of 0.88 (95% confidence interval [CI] = 0.83–0.94) and 0.87 (95% CI = 0.81–0.93) in a 3-year follow-up. In a 5-year follow-up, such patients had respective ORs for PD of 0.94 (95% CI = 0.90–0.98) and 0.93 (95% CI = 0.89–0.98). Patients with T2DM who received ≥300 cDDD of metformin or used metformin with intensity of ≥10 DDD/month experienced no neuroprotective effects after 3 or 5 years. Conclusions. Metformin was associated with PD odds in T2DM in a dose–response association manner. Patients who received low dosage and intensity of metformin use were associated with lower odds of PD, while higher dosage and intensity of metformin use had no neuroprotective effect.

## 1. Introduction

Aging often leads to an increased risk of neurodegenerative diseases (NDs), such as cognitive dysfunction and Parkinson’s disease (PD). PD is the second most common ND to occur with aging and is the most common movement disorder worldwide [1]. The neuropathology of PD is complex and characterized mainly by two features: the progressive loss of dopaminergic neurons in the substantia nigra pars compacta leading to a dopamine deficit in the striatum [2] and the abnormal accumulation and aggregation of alpha-synuclein in Lewy bodies [3].

An increasing body of evidence suggests that both PD and dementia are associated with insulin resistance, which is considered a modifiable risk factor [4]. Emerging evidence supports an association between PD and type 2 diabetes mellitus (T2DM) [5], and patients with T2DM were reported to be at an increased risk of PD, compared with the general population [6]. Metformin is the first-line medication for patients with T2DM, and evidence suggests that metformin can slow aging and reduce the incidence of aging-related diseases [7]. Metformin plays a key role in neuroprotection because it mediates the inhibition of inflammatory responses [8] and slows cognitive decline [9]. Metformin is often used because of its potential ability to slow aging, and it has been reported to have potential in PD treatment [10].

Patients with T2DM may have an increased risk of PD and experience faster PD progression [11]. Studies have demonstrated that patients with T2DM who receive metformin have a lower risk of PD than other patients with T2DM have. Several mechanisms that may underly this association between metformin use and the risk of PD have been proposed [10,12,13,14]. However, studies have also suggested that metformin use may increase the risk of PD [15,16,17], especially in patients with T2DM receiving high doses for a long period [18,19]. Therefore, the association between patients with T2DM receiving metformin and the risk of PD must be clarified. However, few epidemiological studies have investigated this association; even fewer have employed a nationwide database. Therefore, we investigated whether metformin use was associated with the risk of PD in patients with T2DM by using data from the Taiwanese National Health Insurance Research Database (NHIRD).

## 2. Materials and Methods

### 2.1. Data Source

This study performed a secondary analysis of data from 2001 to 2018 from the Longitudinal Health Insurance Database (LHID), a data set released by the Health and Welfare Data Science Center (HWDC) of Taiwan’s Ministry of Health and Welfare. Taiwan established the government-run, single-payer NHI program in 1995. The program covers over 99% of Taiwan’s approximately 23 million residents and has contracts with more than 20,000 medical care facilities, including hospitals, clinics, pharmacies, and medical laboratories, amounting to over 93% of the health-care facilities in Taiwan. Data in the LHID are anonymous; the HWDC uses scrambled, random identification numbers to protect the insureds’ privacy. Therefore, the requirement of informed consent was waived.

### 2.2. Ethical Approval

This study was conducted in accordance with the Declaration of Helsinki. The study protocol was approved by the Central Regional Research Ethics Committee of China Medical University (No. CRREC-109-001).

### 2.3. Study Sample

**Figure 1:**
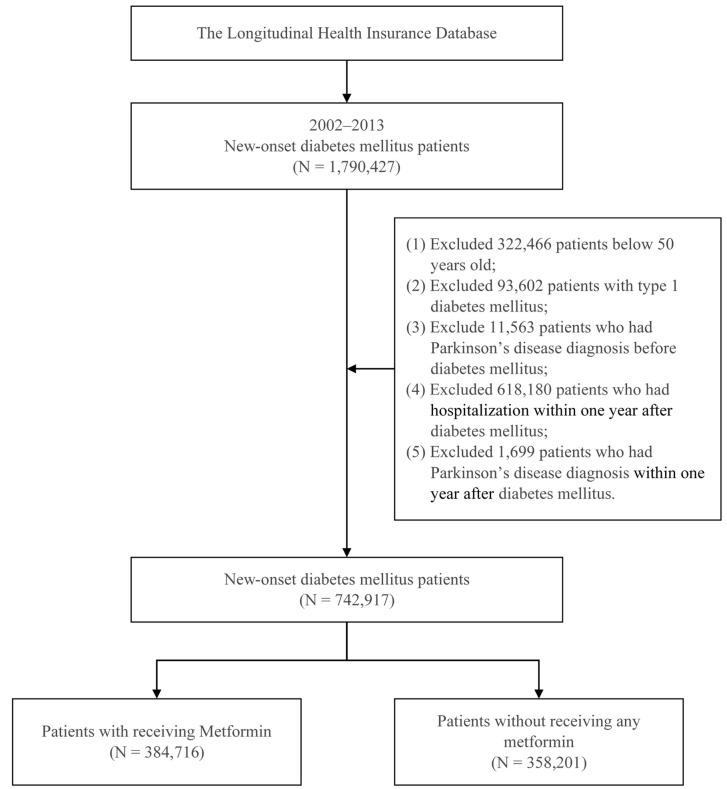
Patient selection process.

To investigate the effects of metformin on PD incidence, we enrolled patients aged over 50 years with new-onset diabetes mellitus from 2002 to 2013. Diabetes mellites was defined as three instances of International Classification of Diseases, Ninth Revision, Clinical Modification (ICD-9-CM) code 250 within a 1-year period. Metformin use was indicated by anatomical therapeutic chemical code A10BA02. To minimize bias, we excluded patients with type 1 diabetes mellitus, a diagnosis of PD before or within 1 year after their diagnosis of T2DM, or any hospitalization within 1 year after their T2DM diagnosis. Patients who had received metformin in the first year after their T2DM diagnosis constituted the case group, and patients who had not received metformin during this period were the comparison group. A total of 742,917 patients with new-onset T2DM between 2002 and 2013 were included. Of these patients, 384,716 did and 358,201 did not receive metformin in the first year after receiving their T2DM diagnosis. The patient selection process is illustrated in Figure 1.

### 2.4. Study Design

This study employed a cross-sectional design and 3-year and 5-year follow-up periods to investigate the risk of PD in patients with T2DM receiving metformin. The defined daily dose (DDD) is a standard measure of drug use and exposure; the World Health Organization defines the DDD as the assumed, average daily maintenance dose of a drug. However, the DDD does not always reflect the recommended or prescribed daily dose [20]. The observation period for each patient’s metformin use was 1 year after T2DM diagnosis. A DDD of 2 g of metformin [21] was used; we calculated the patients’ total metformin exposure for the first year and categorized it as nonuse or <300, 300–500, or >500 cumulative defined daily doses (cDDD) for dose–response analysis. In addition, we calculated the patients’ average monthly doses of metformin and classified them as nonuse or <10, 10–25, or >25 DDD/month to investigate the association between the intensity of metformin use and PD. All of the patients were followed for 5 years. PD was defined as three or more outpatient visit records within 1 year with ICD-9-CM code 332 or International Classification of Disease, Tenth Revision, Clinical Modification (ICD-10-CM) code G20. Comorbid hypertension (ICD-9-CM code 401–405), hyperlipidemia (ICD-9-CM code 272.0–272.4), hyperuricemia (ICD-9-CM 790.6), cerebrovascular disease (ICD-9-CM code 430–438), coronary artery disease (CAD; ICD-9-CM code 414.0), arrhythmia (ICD-9-CM code 427), heart failure (ICD-9-CM code 428.0), anxiety (ICD-9-CM code 300.0), depression (ICD-9-CM code 311), chronic obstructive pulmonary disease (COPD; ICD-9-CM code 490–492 or 494–496), chronic kidney disease (CKD; ICD-9-CM code 585), obesity (ICD-9-CM code 278.00), and alcoholism (ICD-9-CM code 303) were analyzed.

### 2.5. Statistical Analyses

All analyses were performed in SAS software version 9.4 (SAS Institute, Cary, NC, USA). The chi-square test was used to evaluate the distributions of the baseline characteristics of the metformin users and nonusers. The risk of PD for the metformin users and nonusers was estimated by multiple logistic regression with adjustment for relevant variables. The results are presented as odds ratios (ORs) with 95% confidence intervals (CIs). Two adjusted models were developed to estimate PD odds in metformin users based on cumulative metformin exposure (cDDD) and intensity of metformin use (DDD/month). Significance was indicated by a *p* value of <0.05.

## 3. Results

### 3.1. Distribution of Patient Characteristics

The mean age was 62.17 ± 8.86 years (50–64 years [64.02%], 65–74 years [24.74%], and >75 years [11.24%]), as presented in Table 1. Among the patients, 48.57% were women, and 51.43% were men. The average age of the metformin users was 61.32 ± 8.47 years). Among all patients, 168,477 (43.79%) had hypertension; 69,406 (18.04%) had hyperlipidemia; 2850 (0.74%) had hyperuricemia; 17,696 (4.60%) had CVD; 14,429 (3.75%) had arrhythmia; 6893 (1.79%) had heart failure; 34,016 (8.84%) had anxiety; 1796 (0.47%) had depression; 20,660 (5.37%) had COPD; 1546 (0.40%) had CKD; 1752 (0.46%) had obesity; and 225 (0.06%) had alcoholism. The distributions of all comorbid diseases except alcoholism differed significantly between the metformin users and nonusers (*p* < 0.001).

### 3.2. Associations between PD and Metformin Use in Patients New-Onset T2DM—3-Year Follow-Up

Table S1 is the distribution of incident PD in T2DM patients. Table 2 presents the incidence of PD after a 3 year-follow up; 3977 patients (0.54%) developed PD in the 3 years after their T2DM diagnosis. The rate of occurring PD at 3 years of among metformin nonusers was 0.62%, and that among users was 0.46%, 0.45%, and 1.27% for those with <300, 300–500, and >500 cDDD, respectively. Regarding intensity of metformin use, the incidence of PD was 0.46%, 0.43%, and 0.48% among user who averaged <10, 10–25, and ≥25 DDD/month, respectively. After 3 years, patients with T2DM who had received <300, 300–500, and >500 cDDD of metformin had ORs for PD of 0.88 (95% CI = 0.83–0.94), 1.09 (95% CI = 0.72–1.65), and 2.59 (95% CI = 0.83–8.03), respectively. Regarding the intensity of metformin use, patients with T2DM averaging <10, 10–25, and ≥25 DDD/month had ORs for PD of 0.87 (95% CI = 0.81–0.93), 0.92 (95% CI = 0.83–1.02), and 1.17 (95% CI = 0.80–1.72), respectively.

For risk factors, adjusted model 1 revealed that patients with T2DM who were 65–74 and ≥75 years old had ORs for PD of 3.95 (95% CI = 3.64–4.29) and 7.01 (95% CI = 6.42–7.65), respectively. Comparing with diabetes patients with 0 point of Diabetes Complications and Severity Index (DCSI) scores, those with higher value of 1 and ≥2 DCSI scores had ORs for PD of 1.16 (95% CI = 1.06–1.26) and 1.37 (95% CI = 6.42–7.65), respectively. Patients with comorbid CVD (OR = 1.55, 95% CI = 1.41–1.71), anxiety (OR = 1.79, 95% CI = 1.65–1.94), depression (OR = 1.93, 95% CI = 1.50–2.48), or COPD (OR = 1.15, 95% CI = 1.04–1.27) had increased odds of developing PD comparing with those without the respective comorbidities. Patients comorbid with hypertension, hyperuricemia, CAD, arrhythmia, CKD, obesity, and alcoholism did not have an associated risk of developing PD.

### 3.3. Associations between PD and Metformin Use in Patients New-Onset T2DM—5-Year Follow-Up

Table 3 presents the incidence of PD after 5 years. After adjustment for relevant variables, patients with T2DM who had received <300, 300–500, and >500 cDDD of metformin were discovered to have ORs for PD of 0.94 (95% CI = 0.90–0.98), 1.01 (95% CI = 0.75–1.35), and 1.24 (95% CI = 0.40–3.83), respectively. Patients averaging <10, 10–25, and >25 DDD/month had ORs for PD of 0.93 (95% CI = 0.89–0.98), 0.97 (95% CI = 0.90–1.04), and 1.02 (95% CI = 0.77–1.35), respectively. Adjusted model 1 also indicated that patients aged 65–74 and ≥75 years had respective ORs for PD of 3.88 (95% CI = 3.67–4.10) and 6.22 (95% CI = 5.86–6.60). Patients with DCSI scores of 1 and ≥2 had respective ORs for PD of 1.16 (95% CI = 1.09–1.22) and 1.35 (95% CI = 1.27–1.43). Among risk factors, comorbid CVD, anxiety, depression, and COPD were associated with greater PD odds, findings consistent with those at 3 years.

## 4. Discussion

Few large-scale epidemiological studies have evaluated the risk of PD among patients with T2DM receiving metformin. In our study, metformin use was associated with PD risk in T2DM in a dose–response association manner. The results suggest that <300 cDDD of metformin and metformin use of <10 DDD/month are associated with lower odds of PD at 3 and 5 years. However, exposure to ≥300 cDDD of metformin and use intensity of ≥10 DDD/month were associated with no such neuroprotective effects. Our findings also revealed that, among patients with T2DM using metformin, being older and having a high DCSI score were associated with greater odds of PD. Furthermore, metformin users living in highly urbanized areas had greater odds of PD in T2DM.

Patients with T2DM have an increased risk of PD and of experience faster PD progression [11]. PD is an ND characterized by progressive loss of dopaminergic neurons in the substantia nigra. Dysfunctional insulin signaling was reported to increase oxidative stress in PD [22], and an animal study demonstrated that chronic insulin resistance was associated with mitochondrial disruption and dopaminergic neuronal degeneration [23]. We discovered that patients with T2DM who had consumed <300 cDDD of metformin or took metformin with an intensity of <10 DDD/month had lower PD odds. Several mechanisms have been proposed by animal and physiological studies to explain the association between metformin use and PD risk. Research indicates that metformin can cross the blood–brain barrier; its concentration in cerebrospinal fluid is approximately 10% of that in plasma [12].

Metformin may have neuroprotective effects in PD. A murine model of PD demonstrated the metformin can reduce alpha-synuclein phosphorylation and aggregation; influence cellular processes associated with age-related conditions, including autophagy and inflammation [10]; and upregulate neurotrophic factors [24]. Adenosine monophosphate–activated protein kinase (AMPK) plays essential roles in the regulation of neuroenergetic metabolic plasticity and in cognitive impairment [25], and AMPK over activation can lead to the accumulation of alpha-synuclein oligomers and a decrease in neurites [13]. Metformin exerts neuroprotective regulatory effects—its main therapeutic effects in PD—through the AMPK signaling pathway [14].

A study demonstrated that 2-year metformin use was associated with a lower incidence of NDs in older patients with T2DM; however, metformin exposure did not significantly affect the risk of developing NDs during the first 2 years [26]. In our study, patients with T2DM who had received ≥300 cDDD of metformin and those who had a metformin use intensity of ≥10 DDD/month experienced no neuroprotective effects. Studies have suggested that metformin use may increase the risk of PD, with some data suggesting that metformin may cause dementia and PD [15,16,17]. Metformin was also found to increase β-amyloid production [15]. Moreover, AMPK activation by metformin has been demonstrated to induce sufficient metabolic stress to induce dendritic spine loss in hippocampal neurons [27,28]. Several studies have reported an association between prolonged metformin use and vitamin-B12-deficiency-associated peripheral neuropathy. *Diabetic* peripheral neuropathy may be indistinguishable from vitamin B12 deficiency and could lead to permanent nerve damage if correction of deficiency is not prompt [29]. Besides, vitamin B12 deficiency is associated with cognitive impairment [29]. A meta-analysis revealed a negative correlation between metformin use and vitamin B12 levels in patients with T2DM [30], and greater cumulative metformin exposure and duration of use were associated with increased risks of vitamin B12 deficiency [18]. Metformin use is associated with increased risk and severity of vitamin B_12_ deficiency in the elderly patients. Patients receiving metformin ≥1500 mg/day for >2 years are particularly at risk [29]. Although metformin can lower the risk of PD [10,12,14], the B12 deficiency associated with long-term use and high doses of metformin may outweigh the neuroprotective effects and thereby increase the risk of PD. Taken together, metformin related vitamin B12 deficiency may counteract the potential benefit of metformin in long-term therapy. Vitamin B12 deficiency play a part role of PD risk in patients receiving metformin with long-term duration and greater dosage. Our study result is consistent with an animal study show that lower dose of metformin (100 mg/kg) ameliorated scopolamine-induced cognitive deficit, while higher dose of metformin had no deleterious effect [31]. However, the actual underlying mechanism between metformin dosage and PD risk remains unclear and should be investigated in the future. Those DM patients taking longer or more intensive treatment with metformin may have more T2DM severity that can offset the neuroprotective effect. 

The DCSI is a tool for predicting the risk of hospitalization and mortality in patients with diabetes mellitus [32]. The adapted DCSI, which assesses seven categories of diabetic complications without severity grading, is a modified version of a risk measure that does not consider laboratory data [32,33]. Patients with diabetes mellitus and high scores on the adapted DCSI reportedly have a higher risk of dementia [34]. Our study indicates that metformin use with a high DCSI score is associated with increased odds of PD in T2DM. Thus, the DCSI may be used as an indicator of PD risk. Our findings also revealed that older patients (particularly those older than 75 years) with T2DM who were receiving metformin had greater odds of PD. This result is consistent with others suggesting that PD risk increases with age [35]. Age is the greatest risk factor for the PD development and progression [36] and causes many cellular processes that predispose individuals to neurodegeneration. Moreover, age-related pathological alterations of cellular function can predispose individuals to PD [37]. Aging increases the risks of both T2DM and vitamin B12 deficiency [38]. Older patients with diabetes mellitus who receive metformin may have a higher risk of PD due to long treatment durations and high doses, which may lead to vitamin B12 deficiency and severe diabetic peripheral neuropathy [19,39]. Moreover, we discovered that, among patients with T2DM, metformin users who lived in areas with a high level of urbanization had greater odds of PD. This result is consistent with those of other studies, which have reported differences in PD prevalence based on region, country, and urbanization level [35,40].

Our results indicate that metformin users with comorbid CVD, anxiety, depression, or COPD have greater odds of PD in T2DM. A large, population-based study demonstrated that most cerebrovascular risk factors are also associated with subsequent PD [41], and PD is positively associated with depression and anxiety [42]. PD-related anxiety or depression may be due to Lewy body deposition in the serotonergic and noradrenergic neurons [43]. Another study discovered a higher risk of PD among patients with anxiety than among those patients without, and more severe anxiety was associated with a greater PD risk [44]. Depression has been reported to be an independent risk factor for and early symptom of PD [45,46]. Moreover, a cohort study revealed that the risk of PD was significantly higher among patients with COPD than it was in the general population [47].

Our study has several strengths; the first is its population-based design. Our sample was drawn from the entire population of Taiwan; thus, our sample is representative. The population-based design also minimized selection bias, which is common in observational studies. Furthermore, the follow-up information from health-care institutions was nearly complete for the entire sample. Second, the characteristics of the database provided sufficient statistical power to investigate the associations between metformin use and PD risk among patients with T2DM. Third, we evaluated the associations at 3 and 5 years; furthermore, metformin exposure was categorized into <300, 300–500, and >500 cDDD, and use intensity was categorized into <10, 10–25, and >25 DDD/month. Fourth, we investigated relevant risk factors (comorbidities).

Our study also has several limitations. First, behavioral data, such as tobacco smoking habits, alcohol consumption, and caffeine intake, and physical activity habits (and associated body mass index), were unavailable. These factors can affect PD development and thus may have affected our findings [48]. Second, the diagnoses of PD and other comorbidities were based solely on ICD-9-CM and ICD-10-CM codes. However, the Taiwanese Bureau of National Health Insurance randomly reviews charts and interviews patients to verify the accuracy of diagnoses. Hospitals reporting outlier charges or practices are audited, and the penalties for malpractice are severe. These processes ensure the validity and accuracy of the NHIRD. Third, the severity of PD and T2DM cannot be determined from ICD-9-CM and ICD-10-CM codes; thus, severity-based subgroup analysis was impossible. For example, the NHIRD does not provide information regarding HbA1c, which is crucial to hyperglycemia management for patients with diabetes mellitus. Therefore, the potential correlation between glycemic control and PD incidence could not be explored. Fourth, DM patients receiving >500 cDDD of metformin were relatively fewer in our study, as the small sample size may lead to bias.

## 5. Conclusions

In conclusion, metformin use was associated with PD risk among T2DM patients in a dose–response association manner. Patients who received low dosage and intensity of metformin use were associated with lower odds of PD, while higher dosage and intensity of metformin use had no neuroprotective effect.

## Figures and Tables

**Table 1:** Baseline characteristics of patients with new-onset diabetes mellitus.

Variables	Total		Metformin				
			Non-Users		Users		*p*-Value
	N	%	N	%	N	%	
Total	742,917	100.00	358,201	48.22	384,716	51.78	
Gender							<0.001
Female	382,065	51.43	189,612	52.93	192,453	50.02	
Male	360,852	48.57	168,589	47.07	192,263	49.98	
Age (year) (Mean ± SD)	62.17 ± 8.86		63.08 ± 9.18		61.32 ± 8.47		<0.001
50–64	475,631	64.02	215,040	60.03	260,591	67.74	
65–74	183,801	24.74	93,963	26.23	89,838	23.35	
≥75	83,485	11.24	49,198	13.73	34,287	8.91	
Income level (NTD) ^a^							<0.001
≤21,000	385,214	51.85	189,933	53.02	195,281	50.76	
21,001–33,000	176,301	23.73	79,224	22.12	97,077	25.23	
≥33,001	181,402	24.42	89,044	24.86	92,358	24.01	
Urbanization ^b^							<0.001
Level 1	205,119	27.61	104,737	29.24	100,382	26.09	
Level 2	240,126	32.32	115,045	32.12	125,081	32.51	
Level 3	115,321	15.52	52,993	14.79	62,328	16.20	
Level 4	104,346	14.05	49,332	13.77	55,014	14.30	
Level 5	17,554	2.36	8569	2.39	8985	2.34	
Level 6	31,709	4.27	14,535	4.06	17,174	4.46	
Level 7	28,742	3.87	12,990	3.63	15,752	4.09	
DCSI score ^c^							<0.001
0	449,726	60.54	212,961	59.45	236,765	61.54	
1	158,619	21.35	76,184	21.27	82,435	21.43	
≥2	134,572	18.11	69,056	19.28	65,516	17.03	
Hypertension							<0.001
No	407,559	54.86	191,320	53.41	216,239	56.21	
Yes	335,358	45.14	166,881	46.59	168,477	43.79	
Hyperlipidemia							<0.001
No	584,466	78.67	269,156	75.14	315,310	81.96	
Yes	158,451	21.33	89,045	24.86	69,406	18.04	
Hyperuricemia							<0.001
No	736,469	99.13	354,603	99.00	381,866	99.26	
Yes	6448	0.87	3598	1.00	2850	0.74	
Cerebrovascular disease							<0.001
No	704,412	94.82	337,392	94.19	367,020	95.40	
Yes	38,505	5.18	20,809	5.81	17,696	4.60	
Coronary artery disease							<0.001
No	678,208	91.29	323,771	90.39	354,437	92.13	
Yes	64,709	8.71	34,430	9.61	30,279	7.87	
Arrhythmia							<0.001
No	711,085	95.72	340,798	95.14	370,287	96.25	
Yes	31,832	4.28	17,403	4.86	14,429	3.75	
Heart failure							<0.001
No	728,751	98.09	350,928	97.97	377,823	98.21	
Yes	14,166	1.91	7273	2.03	6893	1.79	
Anxiety							<0.001
No	669,132	90.07	318,432	88.90	350,700	91.16	
Yes	73,785	9.93	39,769	11.10	34,016	8.84	
Depression							<0.001
No	739,016	99.47	356,096	99.41	382,920	99.53	
Yes	3901	0.53	2105	0.59	1796	0.47	
COPD ^c^							<0.001
No	697,869	93.94	333,813	93.19	364,056	94.63	
Yes	45,048	6.06	24,388	6.81	20,660	5.37	
Chronic kidney disease							<0.001
No	736,925	99.19	353,755	98.76	383,170	99.60	
Yes	5992	0.81	4446	1.24	1546	0.40	
Obesity							0.008
No	739,680	99.56	356,716	99.59	382,964	99.54	
Yes	3237	0.44	1485	0.41	1752	0.46	
Alcoholism							0.824
No	742,478	99.94	357,987	99.94	384,491	99.94	
Yes	439	0.06	214	0.06	225	0.06	

^a^ The premium-based salary of the patient which is according to the payroll bracket table of the National Health Insurance Administration Taiwan. NTD is New Taiwan Dollar. NTD 1 ≈ USD 0.034). ^b^ Level 1 denoted the highest degree of urbanization, whereas level 7 denoted the lowest degree of urbanization. ^c^ Abbreviations: DCSI, diabetes complications severity index; COPD, chronic obstructive pulmonary disease.

**Table 2:** Three-year follow-up of incident Parkinson’s disease in new-onset diabetes mellitus patients with metformin medication.

Variables	Three-Year Follow-Up							
	Events	%	Adjusted Model 1			Adjusted Model 2		
			OR	95% CI	*p*-Value	OR	95% CI	*p*-Value
Total	3977	0.54						
cDDD of metformin use								
Non-users	2223	0.62	1					
<300	1728	0.46	0.88	0.83–0.94	<0.001	-	-	-
300–500	23	0.45	1.09	0.72–1.65	0.676	-	-	-
≥500	3	1.27	2.59	0.83–8.03	0.100	-	-	-
Intensity of metformin use								
Non-users	2223	0.62				1		
<10	1292	0.46	-	-	-	0.87	0.81–0.93	<0.001
10–25	436	0.43	-	-	-	0.92	0.83–1.02	0.127
≥25	26	0.48	-	-	-	1.17	0.80–1.72	0.426
Gender								
Female	2078	0.54	1			1		
Male	1899	0.53	1.04	0.98–1.11	0.205	1.04	0.98–1.11	0.215
Age (year)								
50–64	940	0.20	1			1		
65–74	1617	0.88	3.95	3.64–4.29	<0.001	3.96	3.64–4.30	<0.001
≥75	1420	1.70	7.01	6.42–7.65	<0.001	7.02	6.43–7.67	<0.001
Income level (NTD) ^a^								
≤21,000	2475	0.64	1			1		
21,001–33,000	803	0.46	0.96	0.88–1.04	0.272	0.96	0.88–1.04	0.274
≥33,001	699	0.39	0.91	0.83–0.99	0.033	0.91	0.83–0.99	0.033
Urbanization ^b^								
Level 1	919	0.45	1			1		
Level 2	1167	0.49	1.09	1.00–1.18	0.066	1.09	1.00–1.18	0.066
Level 3	599	0.52	1.09	0.98–1.21	0.098	1.09	0.98–1.21	0.098
Level 4	712	0.68	1.25	1.13–1.38	<0.001	1.25	1.13–1.38	<0.001
Level 5	169	0.96	1.47	1.25–1.74	<0.001	1.47	1.25–1.74	<0.001
Level 6	230	0.73	1.20	1.04–1.39	0.014	1.20	1.04–1.39	0.013
Level 7	181	0.63	1.10	0.94–1.29	0.239	1.10	0.94–1.30	0.236
DCSI score ^c^								
0	1735	0.39	1			1		
1	890	0.56	1.16	1.06–1.26	<0.001	1.16	1.06–1.26	<0.001
≥2	1352	1.00	1.49	1.37–1.63	<0.001	1.49	1.37–1.63	<0.001
Hypertension								
No	1747	0.43	1			1		
Yes	2230	0.66	0.98	0.91–1.05	0.531	0.98	0.91–1.05	0.529
Hyperlipidemia								
No	3107	0.53	1			1		
Yes	870	0.55	0.90	0.83–0.98	0.010	0.90	0.83–0.98	0.010
Hyperuricemia								
No	3931	0.53	1			1		
Yes	46	0.71	1.12	0.84–1.50	0.436	1.12	0.84–1.50	0.435
Cerebrovascular disease								
No	3398	0.48	1			1		
Yes	579	1.50	1.55	1.41–1.71	<0.001	1.55	1.41–1.71	<0.001
Coronary artery disease								
No	3376	0.50	1			1		
Yes	601	0.93	1.05	0.95–1.15	0.351	1.05	0.95–1.15	0.351
Arrhythmia								
No	3683	0.52	1			1		
Yes	294	0.92	1.01	0.89–1.14	0.909	1.01	0.89–1.14	0.905
Heart failure								
No	3831	0.53	1			1		
Yes	146	1.03	0.83	0.70–0.99	0.039	0.83	0.70–0.99	0.039
Anxiety								
No	3208	0.48	1			1		
Yes	769	1.04	1.79	1.65–1.94	<0.001	1.79	1.65–1.94	<0.001
Depression								
No	3914	0.53	1			1		
Yes	63	1.61	1.93	1.50–2.48	<0.001	1.93	1.50–2.48	<0.001
COPD ^c^								
No	3527	0.51	1			1		
Yes	450	1.00	1.15	1.04–1.27	0.006	1.15	1.04–1.27	0.006
Chronic kidney disease								
No	3897	0.53	1			1		
Yes	80	1.34	1.19	0.95–1.49	0.142	1.19	0.95–1.49	0.140
Obesity								
No	3963	0.54	1			1		
Yes	14	0.43	1.09	0.64–1.84	0.761	1.09	0.64–1.84	0.759
Alcoholism								
No	3974	0.54	1			1		
Yes	3	0.68	1.51	0.49–4.69	0.475	1.51	0.49–4.69	0.476

^a^ The premium-based salary of the patient which is according to the payroll bracket table of the National Health Insurance Administration Taiwan. NTD is New Taiwan Dollar. NTD 1 ≈ USD 0.034). ^b^ Level 1 denoted the highest degree of urbanization, whereas level 7 denoted the lowest degree of urbanization. ^c^ Abbreviations: DCSI, diabetes complications severity index; COPD, chronic obstructive pulmonary disease.

**Table 3:** Five-year follow-up of incident Parkinson’s disease in new-onset diabetes mellitus patients with metformin medication.

Variables	Five-Year Follow-Up of Incident Parkinson’s Disease							
	Events	%	Adjusted Model 1			Adjusted Model 2		
			OR	95% CI	*p*-Value	OR	95% CI	*p*-Value
Total	8488	1.14						
cDDD of metformin use								
Non-users	4584	1.28	1					
<300	3856	1.02	0.94	0.90–0.98	0.006	-	-	-
300–500	45	0.88	1.01	0.75–1.35	0.969	-	-	-
≥500	3	1.27	1.24	0.40–3.86	0.706	-	-	-
Intensity of metformin use								
Non-users	4584	1.28				1		
<10	2890	1.04	-	-	-	0.93	0.89–0.98	0.003
10–25	966	0.96	-	-	-	0.97	0.90–1.04	0.365
≥25	48	0.90	-	-	-	1.02	0.77–1.35	0.900
Gender								
Female	4463	1.17	1			1		
Male	4025	1.12	1.03	0.99–1.08	0.138	1.03	0.99–1.08	0.144
Age (year)								
50–64	2161	0.45	1			1		
65–74	3560	1.94	3.88	3.67–4.10	<0.001	3.88	3.67–4.10	<0.001
≥75	2767	3.31	6.22	5.86–6.60	<0.001	6.22	5.86–6.61	<0.001
Income level (NTD) ^a^								
≤21,000	5239	1.36	1			1		
21,001–33,000	1728	0.98	0.96	0.90–1.01	0.099	0.96	0.90–1.01	0.101
≥33,001	1521	0.84	0.90	0.84–0.95	<0.001	0.90	0.84–0.95	<0.001
Urbanization ^b^								
Level 1	2093	1.02	1			1		
Level 2	2466	1.03	1.01	0.95–1.07	0.879	1.01	0.95–1.07	0.878
Level 3	1297	1.12	1.04	0.97–1.11	0.323	1.04	0.97–1.11	0.322
Level 4	1453	1.39	1.12	1.05–1.20	<0.001	1.12	1.05–1.20	<0.001
Level 5	308	1.75	1.19	1.06–1.34	0.005	1.19	1.06–1.35	0.004
Level 6	482	1.52	1.11	1.01–1.23	0.040	1.11	1.01–1.23	0.039
Level 7	389	1.35	1.04	0.94–1.16	0.456	1.04	0.94–1.16	0.452
DCSI score ^c^								
0	3889	0.86	1			1		
1	1970	1.24	1.16	1.09–1.22	<0.001	1.16	1.09–1.22	<0.001
≥2	2629	1.95	1.35	1.27–1.43	<0.001	1.35	1.27–1.43	<0.001
Hypertension								
No	3785	0.93	1			1		
Yes	4703	1.40	1.00	0.95–1.04	0.818	1.00	0.95–1.04	0.816
Hyperlipidemia								
No	6633	1.13	1			1		
Yes	1855	1.17	0.91	0.86–0.96	<0.001	0.91	0.86–0.96	<0.001
Hyperuricemia								
No	8402	1.14	1			1		
Yes	86	1.33	1.01	0.81–1.25	0.944	1.01	0.81–1.25	0.941
Cerebrovascular disease								
No	7375	1.05	1			1		
Yes	1113	2.89	1.50	1.40–1.61	<0.001	1.50	1.40–1.61	<0.001
Coronary artery disease								
No	7259	1.07	1			1		
Yes	1229	1.90	1.04	0.97–1.11	0.249	1.04	0.97–1.11	0.250
Arrhythmia								
No	7856	1.10	1			1		
Yes	632	1.99	1.08	0.99–1.17	0.073	1.08	0.99–1.17	0.073
Heart failure								
No	8193	1.12	1			1		
Yes	295	2.08	0.87	0.77–0.98	0.027	0.87	0.77–0.98	0.026
Anxiety								
No	6917	1.03	1			1		
Yes	1571	2.13	1.74	1.64–1.84	<0.001	1.74	1.64–1.84	<0.001
Depression								
No	8376	1.13	1			1		
Yes	112	2.87	1.67	1.38–2.01	<0.001	1.67	1.38–2.01	<0.001
COPD ^c^								
No	7583	1.09	1			1		
Yes	905	2.01	1.13	1.05–1.21	<0.001	1.13	1.05–1.21	<0.001
Chronic kidney disease								
No	8351	1.13	1			1		
Yes	137	2.29	1.06	0.89–1.26	0.502	1.06	0.89–1.26	0.499
Obesity								
No	8465	1.14	1			1		
Yes	23	0.71	0.81	0.54–1.22	0.314	0.81	0.54–1.22	0.313
Alcoholism								
No	8483	1.14	1			1		
Yes	5	1.14	1.18	0.49–2.83	0.718	1.18	0.49–2.83	0.717

^a^ The premium-based salary of the patient which is according to the payroll bracket table of the National Health Insurance Administration Taiwan. NTD is New Taiwan Dollar. NTD 1 ≈ USD 0.034). ^b^ Level 1 denoted the highest degree of urbanization, whereas level 7 denoted the lowest degree of urbanization. ^c^ Abbreviations: DCSI, diabetes complications severity index; COPD, chronic obstructive pulmonary disease.

## Data Availability

The National Health Insurance Database used to support the findings of this study were provided by the Health and Welfare Data Science Center, Ministry of Health and Welfare (HWDC, MOHW) under license and so cannot be made freely available. Requests for access to these data should be made to HWDC (https://dep.mohw.gov.tw/dos/np-2497-113.html) (accessed on 6 April 2022).

## Supplementary Materials

The following supporting information can be downloaded at: https://www.mdpi.com/article/10.3390/pharmaceutics14050946/s1, Table S1: The distribution of incident Parkinson’s disease in new-onset diabetes mellitus patients.
