# Supplementary material for: Dose–Response Association of Metformin with Parkinson’s Disease Odds in Type 2 Diabetes Mellitus
Source: Pharmaceutics. 2022 Apr 27;14(5):946. doi: 10.3390/pharmaceutics14050946 (PMC9147745; doi:10.3390/pharmaceutics14050946)
Supplement: Supplementary file 1 [file pharmaceutics-14-00946-s001.zip › pharmaceutics-1692536-supplementary.pdf]

Supplement Table S1. The distribution of incident Parkinson's disease in new-onset diabetes mellitus patients.

Variables	Three-year follow-up					Five-year follow-up				
	Without		With		p-value	Without		With		p-value
	N	%	N	%		N	%	N	%	
Total	738,940	99.46	3,977	0.54		734,429	98.86	8,488	1.14	
cDDD of metformin use					<0.001					<0.001
Non-users	355,978	99.38	2,223	0.62		353,617	98.72	4,584	1.28	
DDD <300	377,627	99.54	1,728	0.46		375,499	98.98	3,856	1.02	
DDD 300-500	5,102	99.55	23	0.45		5,080	99.12	45	0.88	
DDD 500+	233	98.73	3	1.27		233	98.73	3	1.27	
Intensity of metformin use					<0.001					<0.001
Non-users	355,978	99.38	2,223	0.62		353,617	98.72	4,584	1.28	
<10	277,169	99.54	1,292	0.46		275,571	98.96	2,890	1.04	
10~25	100,458	99.57	436	0.43		99,928	99.04	966	0.96	
25+	5,335	99.52	26	0.48		5,313	99.10	48	0.90	
Gender					0.298					0.033
Female	379,987	99.46	2,078	0.54		377,602	98.83	4,463	1.17	
Male	358,953	99.47	1,899	0.53		356,827	98.88	4,025	1.12	
Age (year)					<0.001					<0.001
50-64	474,691	99.80	940	0.20		473,470	99.55	2,161	0.45	
65-74	182,184	99.12	1,617	0.88		180,241	98.06	3,560	1.94	
75+	82,065	98.30	1,420	1.70		80,718	96.69	2,767	3.31	
Income level					<0.001					<0.001
≤21,000	382,739	99.36	2,475	0.64		379,975	98.64	5,239	1.36	
21,001-33,000	175,498	99.54	803	0.46		174,573	99.02	1,728	0.98	
≥33,001	180,703	99.61	699	0.39		179,881	99.16	1,521	0.84	
Urbanization					<0.001					<0.001
Level 1	204,200	99.55	919	0.45		203,026	98.98	2,093	1.02	
Level 2	238,959	99.51	1,167	0.49		237,660	98.97	2,466	1.03	
Level 3	114,722	99.48	599	0.52		114,024	98.88	1,297	1.12	
Level 4	103,634	99.32	712	0.68		102,893	98.61	1,453	1.39	
Level 5	17,385	99.04	169	0.96		17,246	98.25	308	1.75	
Level 6	31,479	99.27	230	0.73		31,227	98.48	482	1.52	
Level 7	28,561	99.37	181	0.63		28,353	98.65	389	1.35	
DCSI score					<0.001					<0.001
0	447,991	99.61	1,735	0.39		445,837	99.14	3,889	0.86	
1	157,729	99.44	890	0.56		156,649	98.76	1,970	1.24	
2+	133,220	99.00	1,352	1.00		131,943	98.05	2,629	1.95	
Hypertension					<0.001					<0.001
No	405,812	99.57	1,747	0.43		403,774	99.07	3,785	0.93	
Yes	333,128	99.34	2,230	0.66		330,655	98.60	4,703	1.40	
Hyperlipidemia					0.398					0.234
No	581,359	99.47	3,107	0.53		577,833	98.87	6,633	1.13	
Yes	157,581	99.45	870	0.55		156,596	98.83	1,855	1.17	
Hyperuricemia					0.049					0.147
No	732,538	99.47	3,931	0.53		728,067	98.86	8,402	1.14	
Yes	6,402	99.29	46	0.71		6,362	98.67	86	1.33	
Cerebrovascular disease					<0.001					<0.001
No	701,014	99.52	3,398	0.48		697,037	98.95	7,375	1.05	
Yes	37,926	98.50	579	1.50		37,392	97.11	1,113	2.89	
Coronary artery disease					<0.001					<0.001
No	674,832	99.50	3,376	0.50		670,949	98.93	7,259	1.07	

Yes	64,108	99.07	601	0.93		63,480	98.10	1,229	1.90	
Arrhythmia					<0.001					<0.001
No	707,402	99.48	3,683	0.52		703,229	98.90	7,856	1.10	
Yes	31,538	99.08	294	0.92		31,200	98.01	632	1.99	
Heart failure					<0.001					<0.001
No	724,920	99.47	3,831	0.53		720,558	98.88	8,193	1.12	
Yes	14,020	98.97	146	1.03		13,871	97.92	295	2.08	
Anxiety					<0.001					<0.001
No	665,924	99.52	3,208	0.48		662,215	98.97	6,917	1.03	
Yes	73,016	98.96	769	1.04		72,214	97.87	1,571	2.13	
Depression					<0.001					<0.001
No	735,102	99.47	3,914	0.53		730,640	98.87	8,376	1.13	
Yes	3,838	98.39	63	1.61		3,789	97.13	112	2.87	
COPD					<0.001					<0.001
No	694,342	99.49	3,527	0.51		690,286	98.91	7,583	1.09	
Yes	44,598	99.00	450	1.00		44,143	97.99	905	2.01	
Chronic kidney disease					<0.001					<0.001
No	733,028	99.47	3,897	0.53		728,574	98.87	8,351	1.13	
Yes	5,912	98.66	80	1.34		5,855	97.71	137	2.29	
Obesity					0.422					0.021
No	735,717	99.46	3,963	0.54		731,215	98.86	8,465	1.14	
Yes	3,223	99.57	14	0.43		3,214	99.29	23	0.71	
Alcoholism					0.671					0.994
No	738,504	99.46	3,974	0.54		733,995	98.86	8,483	1.14	
Yes	436	99.32	3	0.68		434	98.86	5	1.14	
